# Management of clinical failure after minimally invasive surgical therapies (MIST) for BPH: repeat MIST versus resection, enucleation or ablation—a narrative review from EAU endourology

**DOI:** 10.1007/s00345-026-06300-9

**Published:** 2026-02-27

**Authors:** Svetlana Bogatova, Andrey Morozov, Vineet Gauhar, David Lifshitz, Roman Sukhanov, Yaron Ehrlich, Murad Asali, German Krupinov, Shay Golan, Bhaskar K. Somani, Thomas R. W. Herrmann, Dmitry Enikeev

**Affiliations:** 1 Institute for Clinical Medicine, Sechenov University, Moscow, Russia; 2 Institute for Urology and Reproductive Health, Sechenov University, Moscow, Russia; 3 Ng Teng Fong General Hospital (NUHS), Singapore, Singapore; 4 Asian Institute of nephrourology (AINU), Chennai, India; 5 Department of Urology, Rabin Medical Center, Petah Tikva, Israel; 6 Faculty of Medical and Health Sciences, Tel Aviv University, Tel Aviv, Israel; 7 Urology Department, Barzilai University Medical Center, Ben Gurion University of The Negev, Beer Sheva, Israel; 8 University Hospital Southampton NHS Foundation Trust, Southampton, UK; 9 Department of Urology, Spital Thurgau AG, Kantonspital Frauenfeld, Frauenfeld, Switzerland; 10 Division of Urology, Department of Surgical Sciences, Stellenbosch University, Western Cape, Stellenbosch, South Africa; 11 Hannover Medical School, Hannover, Germany; 12 Department of Urology, Medical University of Vienna, Währinger Gürtel 18-20, Vienna, 1090 Austria

**Keywords:** Minimally invasive surgical therapies (MIST), Prostate artery embolization (PAE), Aquablation, Water vapor thermal therapy (WVTT,, Rezum), Prostatic urethral lift (PUL), ITIND (temporary implantable nitinol device), Retreatment, Benign prostate hyperplasia (BPH)

## Abstract

**Introduction:**

Minimally invasive surgical therapies (MIST) for benign prostatic hyperplasia (BPH) have an advantage over other treatment options as they preserve sexual function and may be performed even in frail patients, often under local anesthesia. However, risks of clinical failure after such procedures may be considerably higher compared to standard modalities, making it important to assess the feasibility of repeat MIST in cases requiring retreatment.

**Materials and methods:**

A literature review was conducted in 2 databases, PubMed (Medline) and Google Scholar, with the following search query: (Aquablation OR PUL OR iTIND OR PAE OR Rezum) AND retreatment. The review only included articles that presented reoperation type (repeat MIST or conventional procedures) and its rate after MIST. Data was extracted and summarized in a tabular form.

**Results:**

Repeat MIST as a form of retreatment is technically feasible and has been reported for all modalities except iTIND (temporary implantable nitinol device) owing to the limited number of clinical trials. However, literature is sparse with detailed information about type of retreatment procedures and their outcomes, and reported rates of retreatment are low and variable: from 0% to 28.6% for PAE, 0–6.7% for Aquablation, 1–6.2% for Rezum, 2–20% for PUL. It is important to determine the exact cause of treatment failure. For persistent lower urinary tract symptoms (LUTS) attributable solely to BPH, such as incomplete treatment because of deviation from surgical protocols or untreated median lobe during the initial MIST, as well as BPH recurrence, repeat MIST may be appropriate if technically feasible. If any concomitant conditions (e.g., urethral stricture, bladder neck sclerosis, necrotic tissue after previous procedure) are suspected, and postoperative cystourethroscopy is indicated, combining diagnostic endoscopy with transurethral resection of the prostate (TURP) may be more reasonable. TURP is also preferred strategy in patients with severe LUTS.

**Conclusion:**

Rates of retreatment and need for ongoing therapy following MIST are generally low, and repeat MIST is feasible. Perhaps the best way to minimize re-intervention is to choose the appropriate primary treatment according to evidence based guidelines and to ensure proper surgical training. In case of retreatment, a surgeon should choose between repeat MIST or conventional approaches depending on the cause of relapse and patient’s preferences (TURP or Aquablation for maximum durability and effectiveness; PUL, Rezum or PAE for minimal invasiveness, preservation of sexual function or reduction of operational risk).

## Introduction

Lower urinary tract symptoms (LUTS) associated with benign prostatic hyperplasia (BPH), especially nocturia, pollakiuria and urinary incontinence, considerably reduces quality of life [1]. Individuals with LUTS may have a significantly higher incidence of depressive symptoms [2]. Modern surgical approaches such as endoscopic enucleation of the prostate (EEP) provide durable elimination of LUTS with a low reoperation rate. Manfredi et al. report 5.9% reoperation rate for ThuLEP during a 10-year follow-up and stable international prostate symptom score (IPSS) during the first 5 years with a subsequent slight worsening of urination [3]. However, conventional techniques often reduce quality of life (QoL) by causing retrograde ejaculation and a number of other complications [4]. Retrograde ejaculation is often not even considered to be a complication, yet from the patients’ perspective, it may be crucial to preserve physiological ejaculation. The need to balance efficacy and safety, both for sexually active men and for frail patients in whom even spinal anaesthesia may pose substantial risk, has driven the development of minimally invasive surgical therapies (MIST). In recent years, MIST has become an intriguing alternative to the gold standards of BPH treatment, transurethral resection (TURP) and EEP. While MIST show commendable safety and preserve sexual function, they may be inferior to EEP in terms of urinary flow improvement and long-term durability [5].

The concept of MIST is aligned with the trend of reducing risks associated with surgical interventions. Therefore, most MIST techniques among them are aimed at ablation of BPH or widening the channel without conventional cutting or tissue removal. The PUL (prostatic urethral lift) procedure uses transprostatic implants (UroLift) that expand the lumen of the prostatic part of the urethra, eliminating compression by prostatic tissue. iTIND (temporary implantable nitinol device) is a temporary tulip-shaped implant made of titanium and nickel alloy. The struts are opened in the prostatic part of the urethra at the 5, 7 and 12 o’clock positions, and the urethral lumen expands as a result of mechanically induced prostate ischemia. The device is extracted after 5–7 days. PAE (prostate artery embolization) similarly acts through ischemia which promotes sclerosis and, as a result, reduces prostate volume. Aquablation and the Rezum procedure are ablative methods and use water-based energy. The former employs a high-velocity, robotically controlled water jet to precisely resect prostatic tissue, while the latter delivers convective water vapour to ablate tissue through thermal energy transfer.

Maintaining a balance between efficacy and risk of complications is paramount when selecting a surgical technique. While reducing invasiveness and preserving ejaculatory function, for example, is reasonable, long-term effectiveness should not be sacrificed in favor of achieving these goals. Indications for MIST and their position among other modalities for primary surgery of BPH is more or less clear, but the tactics of the relapse management after the initial MIST have not yet been definitively determined. This narrative review aims to determine whether MIST failure justifies a repeat MIST, or a standard approach (TURP or EEP) is preferable in such a case.

## Materials and methods

The current literature search for this comprehensive narrative review was conducted across the PubMed and Google Scholar databases with the following search query: (Aquablation OR PUL OR iTIND OR PAE OR Rezum) AND retreatment. This research focuses on surgical retreatment after initial MIST and explores the feasibility and outcomes of repeated MIST. The PICOS (Patient Intervention Comparison Outcome Study) framework was used to describe the study concept.

P (patients) – patients with benign prostatic hyperplasia (BPH);

I (interventions) – retreatment after minimally invasive surgery: Aquablation, PAE, PUL, iTIND, Rezum;

C (comparators) – repeat MIST or conventional modalities (TURP, EEP) as a retreatment;

O (outcomes) – retreatment rate and its outcomes (feasibility, efficacy, safety);

S (studies) – all kinds of original studies.

Only articles published in English were accepted for further analysis. There were no publication time restrictions. The articles referenced in each study were also reviewed.

Inclusion criteria: articles that specify not only the retreatment rate, but also the structure of retreatment surgical procedures after MIST and/or other standard surgical treatment.

Exclusion criteria: prostate diseases other than BPH, malignancies, information in the articles only about the results of first-line surgical treatment without mentioning retreatment.

All the data obtained were combined into Table 1.

**Table 1:** Rate and type of retreatment after different MISTs

Author, title, LE	Patients	Surgery	Hospital stay (HS) and catheterization time (CT)	Redo or clinical failures	Drug therapy
Pisco, 2011, 2b Prostatic arterial embolization to treat benign prostatic hyperplasia [38]	15 patients Mean age 74.1 (range 62–82), years	PAE successful in 14/15 patients (93.3%). In one patient (6.7%), the procedure was impossible as a result of tortuosity and atherosclerotic changes of the iliac arteries	HS 12 patients – 6–8 h after the procedure, 3 patients – 18 h. CT 4 patients – 5 days after the procedure, 3 patients – 10 days	4 (28.6%) clinical failures: 3 patients – persisting severe symptoms after PAE and 1 patient – area of ischemia at the bladder base and persisting LUTS	All remaining patients stopped all prostatic medication
Woo, 2011, 2b Safety and feasibility of the prostatic urethral lift: a novel, minimally invasive treatment for lower urinary tract symptoms (LUTS) secondary to benign prostatic hyperplasia (BPH)[8]	19 patients Mean age 66 ± 6 (range 55–77) years	PUL	6 (32%) patients - no postoperative catheter. CT: 11 (58%) - overnight; 1–11 days until he elected to undergo TURP; 1–6 days	3 (16%) TURP at 1-year follow-up: one at 11 days, one at 7 months, and one at 11 months	–
Chin, 2012, 2b Prostatic Urethral Lift: Two-year Results After Treatment for Lower Urinary Tract Symptoms Secondary to Benign Prostatic Hyperplasia[27]	64 patients, Mean age 66.9 ± 7.3 (range 53–83) years	PUL	47% of the patients without postoperative catheterization. Median CT – 20 h, 75% of catheters were removed the day after the procedure.	13 (20%) – TURP, photoselective vaporization of the prostate, or repeat PUL at the 2-year follow-up	–
Pisco, 2013, 2b Embolisation of prostatic arteries as treatment of moderate to severe lower urinary symptoms (LUTS) secondary to benign hyperplasia: results of short- and mid-term follow-up[39]	255 patients Mean age 65.5 ± 7.4 (range 45- 85) years	PAE was technically successful in 250/255 (98%) 5 patients (2%) - the procedure was impossible (tortuosity and atherosclerotic changes of the iliac arteries)	HS 220 patients (88%) – 3–8 h, 30 patients (12%) − 18 h	56 patients – clinical failures. A bladder mass was removed from the patient during simple surgery 1 month later.	–
Carnevale, 2013, 2b Quality of Life and clinical symptom improvement support Prostatic Artery Embolization for patients with acute urinary retention caused by benign prostatic hyperplasia[19]	11 patients Mean age 68.5 ± 5.2 years	PAE		Clinical success (i.e., Foley catheter removal) was observed in 10 patients (91%). 1 patient underwent bilateral embolization twice after failure to urinate following Foley catheter removal in 1 month after initial PAE.	–
Gao, 2014, 1b Benign Prostatic hyperplasia: Prostatic Arterial Embolization versus Transurethral Resection of the prostate – a prospective, randomized, and controlled clinical trial[13]	114 patients in total: 57 PAE Mean age 67.7 ± 8.7 years 57 TURP Mean age 66.4 ± 7.8 years	PAE successful in 54/57 patients (94.7%). In 3 patients (5.3%), PAE was impossible due to tortuosity and atherosclerotic changes of the bilateral iliac arteries	Patients with urethral catheter − 19 (35.2%) Patients with hospital stay 26 (48.1%)	5 (9.4%) clinical failure	–
		TURP	Patients with urethral catheter − 53 (100%) Patients with hospital stay − 53 (100%)	Successful in all 53 patients (100%)	–
Bach, 2014, 2b First Multi-Center all-comers study for the Aquablation Procedure[23]	178 patients Mean age 67.7 ± 8.5 (range 38–88) years	Aquablation	Median CT 1.9 days Mean HS 2.2 days (range 0–12)	No repeat procedures for treatment failure. 1 rectal perforation requiring a temporary colostomy. 3 patients had meatal stenosis or stricture	–
Porpiglia, 2015, 2b Temporary implantable nitinol device (TIND): a novel, minimally invasive treatment for relief of lower urinary tract symptoms (LUTS) related to benign prostatic hyperplasia (BPH): feasibility, safety and functional results at 1 year of follow-up[10]	32 patients Mean age 69.4 ± 8.2 years	iTIND		One patient (3.1%) reported urinary incontinence 1 day after surgery, prompting immediate removal of the device.	No patients required medical therapy
Cantwell, 2014, 1b Multicentre prospective crossover study of the ‘prostatic urethral lift’ for the treatment of lower urinary tract symptoms secondary to benign prostatic hyperplasia[40]	53 patients Mean age 64 ± 8.0 (range 50–79) years	PUL	27 (66%) - no postoperative catheterization was required Mean CT − 33 h	1 patient – TURP 12 months after treatment due to persistent nocturia	–
Dixon, 2015, 4 Efficacy and Safety of Rezum System Water Vapor Treatment for Lower Urinary Tract Symptoms Secondary to Benign Prostatic Hyperplasia[41]	65 patients Mean age 66.6 ± 7.7 years	Rezum	36 patients – catheterized immediately after the procedure or before discharge (mean CT, 5.6 days; range, 1.0–29.1.0.1 days 11 patients – catheterized after discharge (mean CT, 4.3 days; range, 0.3–17.0 days	1 (1.5%) – TURP at 42 days	–
Gilling, 2015, 2b Aquablation– image-guided robot-assisted waterjet ablation of the prostate: initial clinical experience [7]	15 patients Mean age 73 ± 7 (range 59–86) years	Aquablation	Most patients were discharged on the day after the procedure, and all but one patient had removal of the catheter within 24 h	1 (6.7%) - secondary Aquablation within 90 days of the first procedure with no further clinical sequelae at the 6-month follow-up	–
Carnevale, 2015, 1b Transurethral Resection of the Prostate (TURP) Versus Original and PErFecTED Prostate Artery Embolization (PAE) Due to Benign Prostatic Hyperplasia (BPH): Preliminary Results of a Single Center, Prospective, Urodynamic-Controlled Analysis[14]	15 patients Mean age 66.4 ± 5.6 (range 55–78) years	TURP	TURP: mean HS of 2.1 days with continuous bladder irrigation for all patients	No recurrence of LUTS	–
	15 patients Mean age 63.5 ± 8.7 (range 46–75) years	Original PAE		2 (13.3%) – retreatment with TURP	–
	15 patients Mean age 60.4 ± 5.2 (range 53–68) years	PErFecTED PAE		No recurrence of LUTS	–
Isaacson, 2016, 2b Prostatic Artery Embolization Using Embosphere Microspheres for Prostates Measuring 80–150 cm^3^: Early Results from a US Trial[20]	12 patients	PAE		1 (8%) – repeated PAE	–
Yu, 2016, 2b Prostate Artery Embolization for Complete Urinary Outflow Obstruction Due to Benign Prostatic Hypertrophy[5]	16 patients (with AUR- study group) Median age 66 (IQR 60.3, 70.3) years	PAE	Successful TWOC (trial without catheter) in 14 patients	2 patients who failed TWOC after PAE underwent TURP	–
Rukstalis, 2016, 2b Two Year Durability after Crossover to the Prostatic Urethral Lift from Randomized, Blinded Sham[43]	51 patients Mean age 64 ± 7.8 (range 50–79) years	PUL		4 (8%) progressed to TURP and 1 subject (2%) required additional PUL implants.	–
Roehrborn, 2016, 1b Convective Radiofrequency Water Vapor Thermal Therapy with Rezūm System: Durable Two-Year Results of Randomized Controlled and Prospective Crossover Studies for Treatment of Lower Urinary Tract Symptoms due to Benign Prostatic Hyperplasia[36]	188 patients	Rezum	Some patients were electively catheterized for an average of 3.6 ± 3.5 days after treatment	Retreatment rate 3.7%. 1 - open prostatectomy, 3 - secondary Rezum, 4 - TURP. 6 of these 8 interventions - removal of the median lobe that was not initially treated	–
Desai, 2018, 4 Aquablation therapy for symptomatic benign prostatic hyperplasia: a single-centre experience in 47 patients[44]	47 patients Mean age 66 (range 50–79) years	Aquablation	Mean HS 3.1 (range 1–8) days Mean CT 1.9 (range 1–11) days	1 (2.1%) - TURP 3 weeks after Aquablation (inability to void, dribbling and hematuria) 2 patients (inability to urinate after catheter removal) - TURP on day 3 after Aquablation	4 (8.5%) patients – alpha-blockers
Ray, 2018, 1b Efficacy and safety of prostate artery embolization for benign prostatic hyperplasia: an observational study and propensity-matched comparison with transurethral resection of the prostate (the UK ROPE study)[15]	216 patients (PAE) 89 patients (TURP)	PAE TURP		11 (5.1%) reoperation at 12 months follow-up. An additional 32 patients (14.8%) were reported as having, or awaiting, a reoperation outside of the 12-month follow-up period. The reoperation rate for PAE 43 (19.9%), for TURP 5 (5.6%)	–
Sievert, 2018, 2b Minimally invasive prostatic urethral lift (PUL) efficacious in TURP candidates: a multicenter German evaluation after 2 years[28]	86 patients Mean age 66.2 ± 11.5 (range 38–85) years	PUL		Total reoperation rate: 11 (12.8%) - persistence of LUTS or increased PVR. 1 patient elected a second PUL procedure. The other 9 underwent TURP. 8/9 experienced symptom resolution without complications, but 1 patient (whose initial PVR was 1600 ml) remained with a significant PVR after TURP - suprapubic catheter.	By 1 st month 57 (66.3%) patients discontinued all LUTS medication
McVary, 2018, 1b Rezum Water Vapor Thermal Therapy for Lower Urinary Tract Symptoms Associated With Benign Prostatic Hyperplasia: 4-Year Results From Randomized Controlled Study[31]	135 patients Mean age 63 ± 7.1 years	Rezum		6 (4.4%) - secondary treatment for LUTS (1 open prostatectomy, 3 plasma-button transurethral vaporization of the prostate, and 2 retreated with the Rezum procedure)	5.2% patients - alpha-blockers No other drugs were used.
Gilling, 2018, 1b WATER: A Double-Blind, Randomized, Controlled Trial of Aquablation vs. Transurethral Resection of the Prostate in Benign Prostatic Hyperplasia[11]	117 patients Mean age 66.0 ± 7.3 years	Aquablation	Mean HS − 1.4 days in each group Mean CT - median of 1 day in each group	1 (1.5%) after TURP – retreatment Aquablation – 100% success	71% patients stopped medical therapy
	65 patients Mean age 65.8 ± 7.2 years	TURP			90% patients stopped medical therapy
Kadner, 2019 Second generation of temporary implantable nitinol device (iTind) in men with LUTS: 2 year results of the MT-02-study[35]	81 patients Median age 65 (range 45.5–84.5) years	iTIND		2 patients – TURP, HOLEP (12 months after iTIND). 1 year after: TURP (5 patients)	–
Gilling, 2020, 1b Three-year outcomes after Aquablation therapy compared to TURP: results from a blinded randomised trial[12]	117 patients Mean age 66 ± 7.3 years	Aquablation		Overall 3-year retreatment 5 (4.3%) in the Aquablation group and 1 (1.5%) in the TURP group (*p* = 0.4219)	9% patients – started on alpha-blockers and 5-ARI after surgery
	67 patients Mean age 65.8 ± 7.2 years	TURP			14% patients – started on alpha-blockers and 5-ARI after surgery
Johnston, 2020, 4 Rezum water vapour therapy: promising early outcomes from the first UK series[34]	210 patients Mean age 66 years	Rezum		2 (1%) – redo Rezum within the first year (due to persistent or deteriorating symptoms)	–
Desai,2020, 2b Aquablation for benign prostatic hyperplasia in large prostate (80–150 cc): 2-year results[42]	101 patients Mean age 67.5 ± 6.6 years	Aquablation	16 patients (16%) had used a urinary catheter within 45 days prior to enrollment	2 (2%) had recurrent BPH symptoms that required surgical retreatment with TURP and HOLEP	–
Abt, 2021, 1b Prostatic Artery Embolisation Versus Transurethral Resection of the Prostate for Benign Prostatic Hyperplasia: 2-yr Outcomes of a Randomised, Open-label, Single-centre Trial[17]	48 patients Mean age 65.7 ± 9.3 years	PAE	Indwelling catheter in 9 (19%) patients	5 (10%) initially treated with TURP and another patient after PAE (2%) required additional surgical procedures	0 patients after PAE – medical treatment
	51 patients Mean age 66.1 ± 9.8 years	TURP	Indwelling catheter in 12 (25%) patients		2 patients –pharmacotherapy
Chin, 2022, 2b Medium-Term Real-World Outcomes of Minimally Invasive Surgical Therapies for Benigh Prostatic Hyperplasia: Water Vapor Thermal Therapy (Rezum) vs. Prostatic Urethral Lift (UroLift) in a High-Volume Urban Academic Center [32]	515	WVTT	Catheter 5.5 ± 4.5 days (range 0–46)	Reoperation rate – 4.4%	37.5% – medication-free Alpha-blockers − 46.6% 5-ARI – 14.3% Antispasmodic – 17.6% PDE-5 inhibitors – 8.1%
	191	PUL	Catheter 3.5 ± 2 days (range 0–16)	Reoperation rate – 10.2%	16.4% – medication-free Alpha-blockers – 38.2% 5-ARI – 15.5% Antispasmodic – 12.7% PDE-5 inhibitors – 7.3%
Oumedjbeur, 2023, 1b Aquablation versus TURP: 5-year outcomes of the WATER randomised clinical trial for prostate volumes 50–80 ml[24]	62 patients Mean age 67.9 ± 6.8 years	Aquablation		Reoperation rate for Aquablation – 1.6% Reoperation rate for TURP – 3.1%	–
	34 patients Mean age 66.4 ± 7.2 years	TURP			
Kaplan, 2023, 1b Retreatment rates and postprocedural complications are higher than expected after BPH surgeries: a US healthcare claims and utilization study[17]	22,629 patients11392 patients 7529 patients 1585 patients	TURP GreenLightPVP PUL Rezum		1-year retreatment rate for TURP 5.3%, GreenLight PVP 5.3%, PUL 5.9%, Rezum WVTT 6.2%	–
Feiertag, 2023 1b Incidence of Surgical Reintervention for Benign Prostatic Hyperplasia Following Prostatic Urethral Lift, Transurethral Resection of the Prostate, and Photoselective Vaporization of the Prostate: A TriNetX Analysis [29]	14,343 patients Age mean 70.1 ± 9.4	PUL	–	Total reintervention rate at 1 year – 5.1%, at 2 year. – 8.2%, at 3 year. – 11.3%, and at 4 year – 16.1%. Reprocedures at 1 year: repeat UroLift (44%), TURP (33.4%), PVP (18.8%), and HoLEP (2.6%). At 4 year: TURP (43.1%), repeat UroLift (33.9%), and PVP).	–
	92,425 patients	TURP	–	Total reintervention rate at 1 year. – 4.6%, at 4 year – 7.5%. Reprocedures at 1 year.: second TURP – 90.3%, PVP – 6%, and TUIP – 1.3%. At 4-yr: TURP – 84.2%, PVP – 9%, TUIP – 2.4%, HoLEP – 1.1%, and UroLift – 1%.	–
	51,439 patients	PVP	–	Total reintervention rate at 1 year. −3.8%, at 4 year. – 7.8%. Reprocedures at the 1-yr: second PVP – 48.8%, TURP – 43.6%, HoLEP – 2.6%, and TUIP – 2%. At 4-yr: TURP – 49%, repeat PVP 40%, HoLEP – 3%, TUIP – 2.4%, and UroLift – 2.3%.	–

## Results

There was a noticeable lack of articles on newer types of MIST (Rezum, iTIND, PUL) as they have only been adopted within the last few years. The small number of published articles may also stem from the lack of equipment for these techniques worldwide. Conversely, the other methods (such as PAE and Aquablation) are much better studied owing to their earlier introduction into clinical practice. PAE is the oldest of these methods, initially developed for haemostasis in various surgical fields, it was later applied in urology for persistent haematuria. The first reports of PAE for lower urinary tract symptoms (LUTS) date back to in 2000 [6]. Aquablation appeared later and has been studied extensively over the last 10 years [7]. Later still, PUL, iTIND and Rezum became available for BPH treatment. The first observational study on PUL was published in 2011 [8]. This method was approved in the US in 2013 and the UK in 2015 [9]. Rezūm™ is even more recent, receiving approval in the USA in 2015 and the UK in 2017, resulting in a smaller evidence base. Initial clinical experience with iTIND was reported in 2015, but despite promising outcomes, its adoption has remained limited [10].

The final number of articles included in this review reflects a clear trend that the volume of published literature on each MIST technique is directly proportional to the length of time it has been in relatively widespread surgical use. The current review included 28 articles in total: 9 of them were devoted to PAE, 9 to Aquablation, 5 to PUL, 4 to Rezum, 2 to iTIND, and 1 article contains data on Rezum and PUL. What is also noteworthy is that some articles compared MIST to TURP as a conventional option for BPH treatment [11–17]. TURP has been known for a long time and is widely available. In case of clinical failures after MIST, TURP was performed quite often as the retreatment option (Table 1). This surgical choice is arguably justified by the fact that TURP is a well-known technique with proven high efficiency, so it is performed frequently.

TURP as a “first-line surgery” is not markedly inferior to EEP in the prostate volume range where MIST are most often performed (30–80 cm³). As a layered resection technique, TURP may offer greater safety than EEP in cases of prostate tissue fibrosis or when identifying the correct anatomical plane is challenging after a prior MIST. Furthermore, because clinical failure typically prompts urethroscopy or cystoscopy, if findings reveal pathology amenable to TURP, there is little justification for pursuing alternative surgical modalities. In addition, TURP is probably the first and main operation that is being mastered by young urologists. MIST require more experience, and of course are not performed by every surgeon.

The largest number of patients was observed in the article by Kaplan et al. [16] – 22,629 patients (in the TURP group). The same article presents the largest retrospective analysis of healthcare data (level of evidence 1b), including 113,392 observations using various methods. The smallest number of patients is observed in quite early studies published in 2011 and 2015: Pisco et al. [39] and Gilling et al. [7] – 15 patients each. The highest reoperation rate was noted in the study by Ray et al. [15] for PAE procedure – 19.9% over a follow-up period of more than 12 months. The lowest reoperation rate among the presented studies was 1.6% over 5 years in the Oumedjbeur et al. [24] article for the Aquablation procedure.

### Retreatment after initial PAE

As mentioned above, higher number of articles were devoted to PAE, as this method has been known for the longest time compared to all other MIST. Data analysis revealed a tendency for clinical failures to decrease over time. For example, a recent study by Carnevale et al. reported clinical success rates of 100% of PErFecTED PAE as well as of TURP, while after original PAE 2/12 patients required TURP [14]. PAE boasts substantial advantages: many articles show a lower number of patients requiring postoperative catheterization. In a study conducted by Gao et al. [13], PAE warranted catheterization in 35.2% of cases only. PAE does not require urethroscopy, which is a notable advantage because prolonged movement of the endoscope can lead to urethral stricture and contracture of the bladder neck [18]. PAE is not without its limitations though, and tortuosity and/or atherosclerotic changes of arteries, as well as contraindications to contrast agent use (which is a necessary component of the surgical intervention) render surgery impossible. Potential complications also require careful consideration, such as bladder neck necrosis, which can cause bladder tamponade, may require additional surgery to remove necrotic tissues. There have been cases of repeated PAE in the articles by Carnevale et al. [19] and Isaacson et al. [20]: 9% and 8% of patients, respectively, underwent the same second MIST. Repeated PAE is feasible in cases of insufficient embolization during the initial intervention and allows for achieving optimal results in patients with BPH. If there are suspicions of urethral stricture, bladder neck sclerosis, necrotic tissues after PAE that obstruct the lumen of the urethra, it is better to start with urethroscopy under general anesthesia to minimize the patient’s discomfort. Furthermore, this approach will allow a quick switch to TURP, which will solve these problems.

### Retreatment after initial aquablation

The second most studied method is Aquablation. It is highly precise method with minimal damage which need general anesthesia. Thus, Aquablation may be classified as a “bridge” technique between standard endoscopic surgeries (TURP, EEP) and MIST [21]. The first publications mentioning this surgical method date back to 2014 [22]. Table 1 revealed a trend similar to PAE and the total reoperation rate decreased over time. However, retreatment rates after Aquablation have never been very high. Two articles [11, 23], report 100% success rates in LUTS treatment with this type of MIST. Some authors [7] reported cases of repeated Aquablation procedures (6.6% of patients underwent a second Aquablation), which makes this surgical option technically possible. The most well-known and fundamental studies of Aquablation known as ‘Water’ also report a very low reoperations rate but do not specify the type of intervention. In Water-1, 5-year reoperation rate was 1.6% [24], in Water-2 this rate was 3% [25], and in Water-3 retreatment was required in 10.7% patients with preoperative prostate volume < 150 cm^3^ and 24.2% with the larger glands [26].

### Retreatment after initial PUL

Current European Association of Urologists (EAU) and American Urological Association (AUA) guidelines recommend PUL as BPH treatment in patients with a prostate volume of 30–80 cm^3^. In general, the authors of the included articles followed this recommendation. In the article by Chin et al. [27], the mean prostate volume was 51 cm^3^ with a range of 21–149 cm^3^. Similar values were reported by Sievert et al. where the mean prostate volume was 43 cm^3^ with a range of 17–111 cm^3^ [28]. Both articles described cases of an additional implant as well as TURP as retreatment after MIST. A major study conducted by Feiertag et al. showed the total reoperation rate after the first PUL was 5.1% (within 1 year after the intervention). Analysis of the retreatment procedures revealed that repeat PUL was most common (44%), followed by TURP (33.4%), photoselective vaporization of the prostate (PVP; 18.8%), and HoLEP (2.6%) [29]. A remarkable note is that PUL in its turn may be used as a retreatment after conventional endoscopic procedures (PVP and TURP), the rate of this approach ranges from 1% to 2.3%.

Repeated PUL is technically reasonable when the prostate volume is sufficient for a second UroLift. The second PUL can be performed if there is insufficient deobstruction [28]. However, clinical failure after PUL in small prostates makes repeating the same MIST an irrational retreatment choice.

PUL can be considered as one of the most preferable modalities for sexually active patients. According to reports by Roehrborn et al. [30], PUL is better than medical therapy as BPH treatment not only preserves but also improves ejaculatory and erectile functions. Patients who underwent PUL showed significant improvement in erectile function by 17% at 12 months (*p* = 0.015) and in ejaculatory function by 35% at 12 months (*p* < 0.0001). By contrast, drug therapy either had no effect or even resulted in a decline of these functions.

### Retreatment after initial rezum

Reoperation rates after Rezum are relatively low (the highest retreatment rate of 4.4% (6 patients) was reported by MacVary et al. [31]). Chin et al. reported a lower retreatment rate comparing Rezum and PUL (4.4% vs. 10.2%) [32]. The need for additional surgery after WVTT (water vapor thermal therapy) is mostly similar to that after PUL: persistent LUTS may be caused by the median lobe, left intact during the first surgery. Initially, it could be regarded as insufficiently hypertrophied tissue or its removal could impair ejaculatory function [33]. Many authors explored the possibility of the repeated Rezum procedure without any technical difficulties. Rezum surgery can effectively reduce the volume of the prostate: Johnston et al. showed an average decrease of 33% [34]. Another noteworthy finding is the absence of de novo erectile dysfunction, which makes this technique suitable for sexually active patients.

### Retreatment after initial iTIND

As for the newest technique, iTIND, in one of the earliest studies conducted in 2014 [10], no cases where reoperation was needed were reported. However, 1 patient (3.1%) had urinary retention, warranting immediate removal of the implant. Kadner et al. associated clinical failure after iTIND with the presence of the median lobe of the prostate, left intact during initial surgery [35]. As a result, patients progressed to TURP (6 patients out of 81) and HoLEP (1 patient).

### Limitation of retreatment analysis

Besides type of MIST, several other factors may influence retreatment rate. Among these factors are age, procedure duration, prostate volume, etc. Unfortunately, obtained data did not allow us to check these interactions. The authors intended to conduct a meta-analysis comparing outcomes of retreatment with TURP or repeat MIST, yet scarce data reported in literature about retreatment outcomes did not allow us to perform such a head-to-head comparison. In general, reported rates of repeat procedures are low which may be caused by bias: some patients in case of relapse may prefer to seek treatment in other centers, thus their retreatment may be not recorded. Reporting bias and short follow-up also may influence retreatment rate provided in literature. The authors who report retreatment usually provide only its rate and don’t specify its type or outcomes. Besides, the samples in majority of studies are small. The core body of evidence is provided by studies of Feiertag et al. [29] with 14,343 cases of PUL and data on retreatment type, and Chin et al. [32] with 515 WVTT and 191 PUL.

The current narrative review has explored the most known and studied methods, but there are also other techniques that have not yet gained their popularity and widespread use due to lack of approval. These modalities include different types of implantable devices and stents of variable shapes [36] (ClearRing™, ZenFlow™ Spring, Butterfly™ and the Urocross Expander System). Once further development brings novel approaches into clinical practice, they will be included in future analyses as well. The authors suggest that the investigators who conduct such procedures report details of repeat surgery: the reason for the repeat procedure, the type of procedure, its features and outcomes.

### Therapy discontinuation after surgery

In the current article, we highlighted the possibility of conducting repeat MIST, as well as a reoperation rate after initial MIST. But besides this, a crucial and little-discussed topic is the rate of continuous medical therapy after the surgery. This topic is explored in the review by Spivak et al. [37]. Many patients elect surgery in order to get rid of medical treatment, and thus if they cannot discontinue therapy, it mismatches their expectations and may considerably worsen quality of life. In our sampling, only minority articles contain information about drug therapy [10–12, 17, 27, 30, 31, 38, 41]. Most of them report the surgical treatment tactics have significantly reduced the use of medications, with proportion of patients requiring therapy ranging from 0 (after PAE and iTIND) up to 29% (Aquablation) and 33% (PUL). Nevertheless, the work of Chin et al. [31] informs about a rather low number of medication-free patients: only 37.5% patients after WVTT and 16.4% after PUL were medication-free. In contrast, the Aquablation trials demonstrated markedly higher rates—98.4% in WATER I [24], 94% in WATER II [25], and 71.2% in WATER III [26]. Of course, this data should be analysed with regard to severity of pre-operative LUTS, follow-up period and indications for therapy.

### The urologist’s tactics

What should a surgeon choose considering for retreatment one of MIST options or standard surgical methods? In case of primary treatment failure, it is logical to follow the direction from a non-invasive methods to a more invasive one. First, it is important to characterize this failure precisely: which symptoms prevail (irritative or obstructive). For this purpose, it is necessary to repeat all the standard test: ask the patient to complete an IPSS questionnaire, fill in a urination diary and undergo uroflowmetry. Next, the urologist needs to evaluate the “substrate” of the current failure. There are different variants: urinary tract infection, urethral stricture, bladder neck contracture, intact middle lobe during the initial MIST, foreign substances (a fragment of the prostate tissue or blood clot), initially large prostate volume (i.e. more than recommended for each particular type of surgery). The patient has to undergo the urinalysis, transrectal ultrasound examination of the prostate (TRUS) and the anamnesis data has to be evaluated as well. Attention should be paid to the time of the LUTS recurrence, whether the symptoms increased or appeared suddenly and the presence of blood in the urine (hematuria). Finally, the transition to direct visualization may be justified: urethrography and urethroscopy.

If any treatment is technically feasible, a patient’s priority is especially important: maximum durability and effectiveness (TURP, Aquablation) or minimal invasiveness, rapid recovery and preservation of ejaculatory function (PAE, Rezum, PUL). Summing up the key features of the repeat procedures, TURP is still considered the most “clear” and predictable operation for BPH. It shows a consistently low retreatment rate: from 0% (in the Gao et al. [13] study) to a maximum of 10% in the article by Abt et al. [17]. However, this surgery is accompanied by 100% catheterization and longer hospitalization in comparative studies (Gao et al. [13]) and it is justified as cost-effective surgery only in patients with severe LUTS.

Aquablation, which is often considered along with MIST, shows a comparable and even lower frequency of repeated surgeries in the long term (1.6% over 5 years) [24]. At the same time, the catheterization and hospitalization time is usually shorter than that of TURP. It is important to mention that the prostate volume for Aquablation is not a significant limitation: in the WATER study [11], Aquablation was performed in patients with a prostate volume up to150 ml, although current clinical guidelines still recommend it in patients with prostate volume of 30–80 ml.

PAE has the highest variation in the reoperation rate parameter (from 0% when performing PErFecTED PAE (Carnevale et al. [14] 2015) to 28.6% (Pisco et al. [39] 2011). But this method is feasible irrespective to prostate volume and does not require anaesthesia. Catheterization is not always required, and the amount of blood loss is less than in the case of TURP. There are anatomical limitations for PAE: tortuosity and atherosclerotic changes of the iliac arteries. The procedure may also be limited in patients with renal insufficiency and thyroid disorders. To resolve the issue of prescribing this operation, EAU guidelines recommend an interdisciplinary approach involving both urologists and radiologists.

PUL has quite rapid recovery (often the catheter is not required, and hospitalization time is minimal). However, the reoperation rate in the medium term (2–4 years) is noticeably higher than that of TURP (10.2% in Chin et al. [32], 2022; 11.3–16.1% in 3–4 years in Feiertag et al. [29], 2023). It often requires continued drug therapy. However, PUL has a low incidence of postoperative sexual disorders, so current EAU recommendations recommend performing PUL in patients with LUTS who are interested in maintaining ejaculatory function with a prostate volume of less than 70 ml without a median lobe.

Rezum demonstrates a low and stable level of repeated interventions after it (up to 6.2% according to Kaplan et al. [16]). The effectiveness is high, and the method is generally not inferior to TURP. Importantly, this option allows to maintain ejaculatory and erectile function, which makes it a preferred alternative to TURP for sexually active patients. Moreover, it is the cost-effective strategy as first-line therapy in patients with moderate LUTS.

It is still very difficult to analyze the results of iTIND, as further long-term studies are needed to objectively assess the effectiveness and retreatment rate after this surgery.

## Conclusion

All MIST can be an adequate alternative for patients with concomitant diseases and those who want to preserve sexual function. Rates of retreatment and need for continuous therapy following MIST are low.

While any method can be repeated, the authors cannot be sure this is also true for iTIND (owing to a lack of data for analysis, as this technique is only available to a limited number of medical centers). Therefore, it is imperative to reveal the real cause of relapse and assess the adequacy of initial MIST: monolateral/bilateral embolization, untreated median lobe, prostate volume larger than recommended for this surgical option, etc. In case of deviation from the standard protocol, it is advisable to complete surgery according to the conventional course of the procedure. Also patient’s preferences should be considered while choosing retreatment: TURP or Aquablation may be preferrable for maximum durability and effectiveness; PUL, Rezum or PAE for minimal invasiveness, preservation of sexual function or reduction of operational risk).

Perhaps the best way to minimize re-intervention is to choose the appropriate primary treatment according to evidence based guidelines and to ensure proper surgical training.

## Data Availability

No datasets were generated or analysed during the current study.
